# Supplementary material for: MetaRibo-Seq measures translation in microbiomes
Source: Nat Commun. 2020 Jun 29;11:3268. doi: 10.1038/s41467-020-17081-z (PMC7324362; doi:10.1038/s41467-020-17081-z)
Supplement: Supplementary file 10 — Supplementary Data 7 [file 41467_2020_17081_MOESM10_ESM.zip › File2/Confidence_High_Taxonomy/108997_out.krona.html]

Javascript must be enabled to view this page.

members
magnitude
magnitudeUnassigned
count
unassigned
taxon
rank

108997\_out

39

superkingdom
2
39

1239
phylum
39

39
186801
class

order
186802
39


SRS142503\_contig\_number\_contig-100\_11721.263259
1
family
186803
38

39491
species

SRS012969\_contig\_number\_38530SRS142890\_contig\_number\_42049SRS147977\_contig\_number\_12246
3

14
genus
1506577

14

SRS014287\_contig\_number\_contig-100\_944.77552SRS020233\_contig\_number\_contig-100\_173.342520SRS023526\_contig\_number\_contig-100\_176.235471SRS044535\_contig\_number\_contig-100\_7225.51615SRS047014\_contig\_number\_contig-100\_14142.194342SRS050422\_contig\_number\_22870SRS051610\_contig\_number\_6504SRS053573\_contig\_number\_2631SRS062427\_contig\_number\_7700SRS074964\_contig\_number\_contig-100\_38.57682SRS077849\_contig\_number\_32396SRS104636\_contig\_number\_29227SRS146764\_contig\_number\_28645SRS893342\_contig\_number\_contig-100\_4357.4358
29361
species

1
1506553
genus

208479
species

SRS148721\_contig\_number\_22288
1

species
1898203
14

SRS014613\_contig\_number\_contig-100\_3045.108504SRS015782\_contig\_number\_28043SRS018836\_contig\_number\_contig-100\_16382.64928SRS019397\_contig\_number\_contig-100\_2320.85706SRS049773\_contig\_number\_contig-100\_18865.68017SRS063985\_contig\_number\_contig-100\_2319.164722SRS077024\_contig\_number\_contig-100\_12887.58596SRS1041032\_contig\_number\_8791SRS1041091\_contig\_number\_18920SRS147139\_contig\_number\_contig-100\_2915.245044SRS148159\_contig\_number\_contig-100\_4819.321074SRS149784\_contig\_number\_contig-100\_33639.75214SRS893252\_contig\_number\_contig-100\_16186.92668SRS893378\_contig\_number\_contig-100\_14577.95638

genus
1407607
4

1806509
species
4

SRS014459\_contig\_number\_32796SRS047044\_contig\_number\_11783SRS050752\_contig\_number\_20643SRS893358\_contig\_number\_1432

1
841
genus

1

SRS015065\_contig\_number\_26046
species
1897016

family
31979
1

1
1485
genus

1896978
species
1

SRS019068\_contig\_number\_106296
